# Novel Dielectric Coagulometer Identifies Hypercoagulability in Patients with a High CHADS_2_ Score without Atrial Fibrillation

**DOI:** 10.1371/journal.pone.0156557

**Published:** 2016-06-08

**Authors:** Yuki Hasegawa, Satomi Hamada, Takuro Nishimura, Takeshi Sasaki, Yusuke Ebana, Mihoko Kawabata, Masahiko Goya, Mitsuaki Isobe, Takatoshi Koyama, Tetsushi Furukawa, Kenzo Hirao, Tetsuo Sasano

**Affiliations:** 1 Department of Biofunctional Informatics, Tokyo Medical and Dental University, Tokyo, Japan; 2 Department of Cardiovascular Medicine, Tokyo Medical and Dental University, Tokyo, Japan; 3 Heart Rhythm Center, Tokyo Medical and Dental University, Tokyo, Japan; 4 Department of Laboratory Molecular Genetics of Hematology, Tokyo Medical and Dental University, Tokyo, Japan; 5 Department of Bio-informational Pharmacology, Medical Research Institute, Tokyo Medical and Dental University, Tokyo, Japan; University of Kansas Medical Center, UNITED STATES

## Abstract

**Background:**

Recent reports showed that the CHADS_2_ score predicted the risk of strokes in patients without atrial fibrillation (AF). Although the hypercoagulability may contribute to the thrombogenesis, it has not been fully investigated due to a lack of a sensitive evaluation modality. Recently a novel dielectric blood coagulometry (DBCM) was invented for evaluating the coagulability by measuring the temporal change in whole blood dielectric permittivity.

**Objective:**

We evaluated the utility of the DBCM for identifying the coagulability.

**Patients/Methods:**

For fundamental experiments, 133 citrated blood samples were drawn from subjects with or without heparin administration. A DBCM analysis was performed to find the adequate coagulation index, and to delineate its measurement range by adding recombinant human tissue factor (TF) or heparin. Then the coagulability was assessed by DBCM and conventional coagulation assays in 84 subjects without AF, who were divided into 3 groups by their CHADS_2_ score. Another 17 patients who received warfarin were also assessed by DBCM to evaluate the effect of anticoagulants.

**Results and Conclusions:**

We calculated the derivative of the dielectric permittivity change after recalcification, and extracted the end of acceleration time (EAT) as a novel index. The EAT showed a dose-dependent shortening with the addition of serial dilutions of TF (×10−^2^ to ×10−^4^), and a dose-dependent prolongation with the addition of heparin (0.05 to 0.15 U/ml). The EAT was significantly shorter in the higher CHADS_2_ score group (19.8 ± 4.8, 18.6 ± 3.1, and 16.3 ± 2.7 min in the CHADS_2_ = 0, 1, and ≥2 groups, respectively, p = 0.0065 by ANOVA). Patients receiving warfarin had a significantly more prolonged EAT than those without warfarin (18.6±4.2 vs. 25.8±7.3 min, p <0.001). DBCM detected the whole blood coagulability with a high sensitivity. Subjects with higher CHADS_2_ scores exhibited hypercoagulability without AF.

## Introduction

Atrial fibrillation (AF) is the most common sustained form of tachyarrhythmias, and it has been widely accepted that AF is an independent risk factor for a stroke [1]. The CHADS_2_ score, or CHA_2_DS_2_-Vasc score are widely utilized for the risk stratification of strokes [2, 3], and used to guide anticoagulation therapy in patients with AF [4]. Although the CHADS_2_ score was developed to target patients with AF, the components of the CHADS_2_ score (congestive heart failure, hypertension, age ≥75, diabetes mellitus [1 point each], and prior strokes or transient ischemic attacks [2 points]) are well known contributors to cardiovascular events, independently of AF. Several findings indicated that a higher CHADS_2_ score was related to a poor prognosis both in patients with and without AF [5]. Further studies revealed that the CHADS_2_ score predicted the risk of strokes in the absence of AF with coronary heart disease [6] and patients without AF [7, 8] including asymptomatic AF [9, 10].

In the classical recognition of the mechanism of thrombosis by Virchow, blood clot formation is accelerated by three factors: the stasis of the blood flow, endothelial injury, and hypercoagulability. It has been considered that the components of the CHADS_2_ score are related to the risk factors for endothelial impairment and atherosclerosis. Moreover, several studies have indicated that aging [11, 12], diabetes [13–15], and heart failure [16, 17] are also involved in the increased coagulability of blood. These findings suggested that a high CHADS_2_ score was related to the hypercoagulability. However, the relationship between the CHADS_2_ score and coagulability of blood has not been fully elucidated.

Another issue regarding the assessment of the coagulability is the small amount of established modalities to quantify the change in the whole blood coagulability. Recently a novel dielectric blood coagulometry (DBCM) has been invented for the evaluation of the coagulability [18, 19]. The DBCM measures the temporal change in the whole blood dielectric permittivity, which represents the aggregation of red blood cells. Although the theoretical studies have been published, a clinically relevant coagulation index has not been established utilizing the DBCM.

We hypothesized that the DBCM may have a potential to delineate small changes in the whole blood coagulability, and may identify the hypercoagulability related to a high CHADS_2_ score. Thus we aimed to establish a novel index to represent the whole blood coagulability from the DBCM analysis, and to compare it among different CHADS_2_ score patients without AF.

## Materials and Methods

### Study subjects

The study group consisted of a cumulative total of 234 subjects including healthy controls and patients who were referred to Tokyo Medical and Dental University for the treatment of cardiovascular disease. Exclusion criteria were as follows; documented AF, recent malignant disease, treatment with anticoagulants or contraceptives, systemic inflammation, and an abnormal bleeding history. The study was approved by the ethics committee of Tokyo Medical and Dental University (No. 1849). Blood samples were collected after written informed consent was obtained.

### Collection of blood samples and conventional coagulation assays

Blood samples were drawn from the cubital vein with minimum stasis unless described specifically. The first 0.5 ml of the drawn blood was discarded, and the remaining blood was kept in tubes containing 3.13% sodium citrate. The collected blood was analyzed by DBCM and conventional coagulation assays. The blood samples were kept at room temperature until the DBCM measurement. The measurement of the DBCM was performed at 3 to 5 hours after the blood collection. For the measurement of the conventional coagulation assays, plasma was obtained by centrifugation with 1500 × g for 15 min at room temperature. The conventional assays were performed with Recombiplastin (Instrumentation Laboratory, MA, USA) for the prothrombin time (PT), Thrombocheck APTT-SLA (Sysmex, Kobe, Japan) for the activated partial thromboplastin time (aPTT), and LPIA genesis D-dimer (LSI Medience, Tokyo, Japan) for the D-dimer.

### Dielectric Blood Coagulometry (DBCM)

DBCM was performed using a prototype dielectric coagulometer (Sony Corp., Tokyo, Japan). The DBCM measured the dielectric permittivity in frequencies ranging from 100 Hz to 16 MHz, with sampling intervals of 1 min. The measurement was completed 60 min after the recalcification. The dielectric permittivity was normalized compared to its initial value, and represented normalized permittivity. The blood samples were heated at 37°C throughout the measurements. The DBCM utilized 180 μl of citrated whole blood. The blood sample was initially mixed with 15 μl of 160 mM CaCl_2_, and other agents if needed. The result of the DBCM was analyzed by conducting a 5-point smoothing derivative of the dielectric permittivity at 10 MHz using the linear/quadratic Savitzky-Golay filter.

This study consisted of 5 experiments: an establishment of a novel coagulation index from the temporal change in the dielectric permittivity (study 1), an evaluation of the reproducibility and the measurement range of the DBCM for detecting the hyper- and hypo-coagulability (study 2, 3, and 4), and an evaluation of the blood coagulability in the patients with cardiovascular disease (study 5).

#### Study 1

Citrated blood samples were obtained from 50 healthy subjects and 10 patients who underwent a cardiac physiological study with the administration of heparin. In the patient group, blood was drawn through an introducer sheath. The normal blood and heparinized blood were analyzed by the DBCM.

#### Study 2

Forty cases were enrolled for an evaluation of the within-run reproducibility of the DBCM from the same blood sample.

#### Study 3

Twenty-five subjects were enrolled for a comparison between the DBCM analysis and activated coagulation time (ACT). The ACT was measured using a Hemochron 401 (International Technidyne Corp., Edison, NJ, USA).

#### Study 4

Eight healthy controls were enrolled in this protocol. A citrated blood sample was taken, followed by a mixing serial (0, 0.05, 0.10, and 0.15 U/ml) concentration of unfractionated heparin (Novo heparin, Mochida Pharmaceutical, Tokyo, Japan). Another set of samples was prepared by adding a recombinant human tissue factor (TF) reagent (Recombiplastin), with a serial dilution ranging from ×10−^2^ to ×10−^6^. Five μl of a diluted TF reagent was added with CaCl_2_ to citrated blood at the beginning of the DBCM analysis. The blood samples with heparin or the TF reagent were analyzed with a control sample by DBCM.

#### Study 5

Eighty-four subjects were enrolled. Citrated blood samples were analyzed by the DBCM. Further, the PT, aPTT, and D-dimer were also measured in a subgroup of subjects. The subjects were classified according to their CHADS_2_ or CHA_2_DS_2_-Vasc score for a comparison of the coagulation parameters. We also enrolled 17 patients who received anticoagulation with warfarin, to compare the DBCM parameters between the groups with and without anticoagulation.

### Statistical analysis

Statistical analyses were performed by JMP^®^10 software (SAS Institute Inc., Cary, NC, USA). The data are expressed as mean ± standard deviation. Two group comparisons were analyzed by the unpaired Student’s t-test or paired t-test. Three group comparisons were analyzed by one-way ANOVA and Tukey-Kramer’s multiple comparison. The relationship between 2 parameters were explored using Pearson correlation test, and intraclass correlation efficient was calculated. A p <0.05 was considered statistically significant.

## Results

### Temporal and Spectral Changes in the Dielectric Permittivity with whole blood coagulation

**Fig 1:**
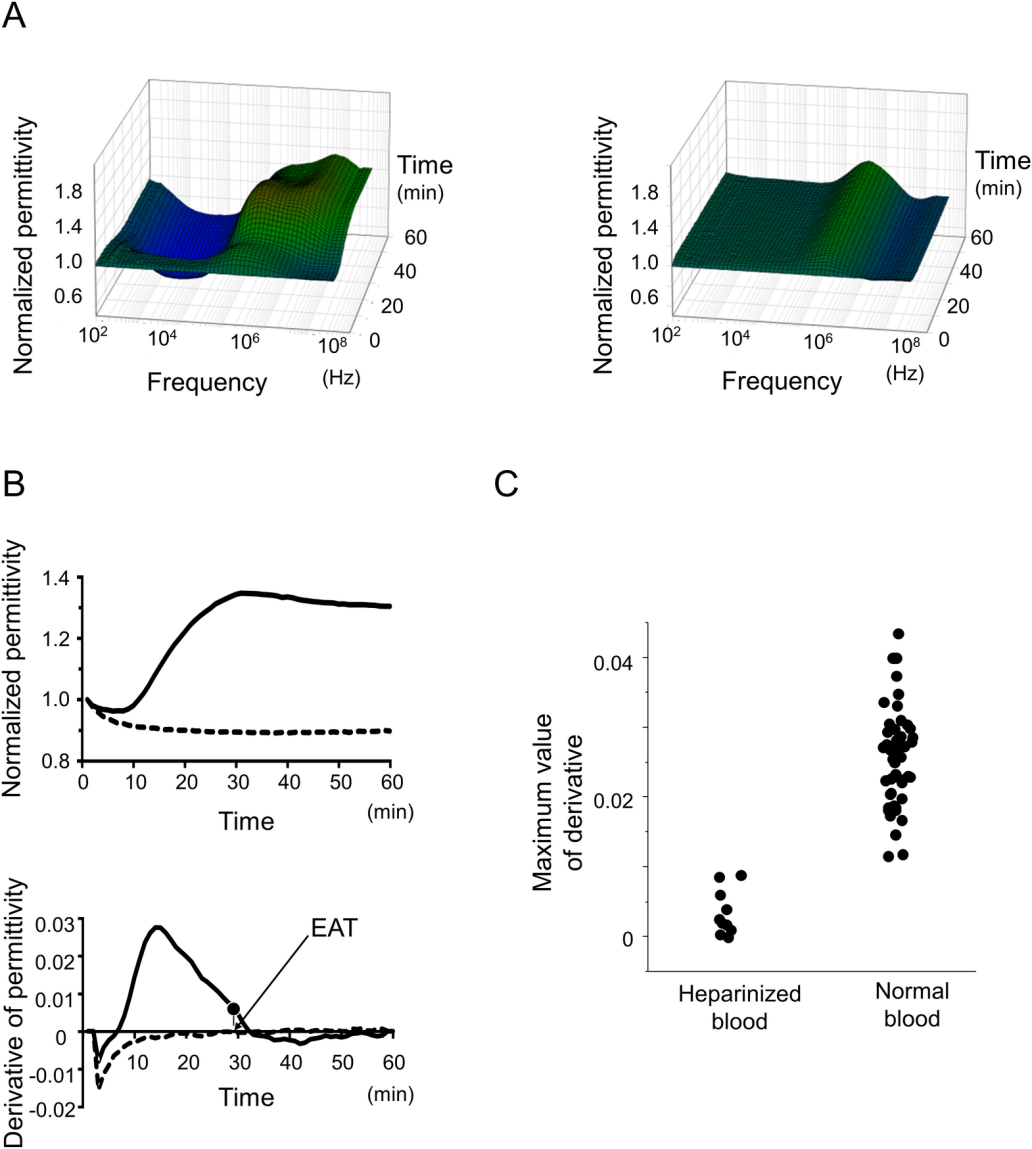
Temporal and spectral changes in the dielectric permittivity. (A) The normalized permittivity after recalcification is plotted from samples with a blood clot formation (left) or without coagulation in patients who underwent an intravenous heparin administration (heparinized samples) (right). The dielectric permittivity gradually increases at a range between 2.5 MHz and 16 MHz in samples with clot formation, whereas heparinized samples show no change in the permittivity in the same frequency range. (B) Representative traces of a normalized permittivity (top) and its derivative (bottom) at 10 MHz plotted against the time from recalcification. The solid line represents the trace from a normal sample with blood clot formation, and the dotted line is that from a heparinized sample without clot formation. The temporal changes in the dielectric permittivity demonstrate a sigmoidal increase, and its derivative shows a single peak in the normal sample. The heparinized sample exhibits no increase in the permittivity, as well as the derivative. The end of acceleration time (EAT) was defined as the time at which the derivative of the permittivity reached the 10% value in the descending phase. (C) The maximum value of the derivative of the dielectric permittivity was plotted in normal samples with a blood clot formation and heparinized sample without clot formation (n = 50 for normals, and 10 for heparinized samples).

We pursued to establish an adequate index to evaluate the coagulation status in the DBCM analyses. The DBCM measured dielectric permittivity in a frequency range from 100 Hz to 16 MHz every 1 minute, up to 60 minutes after recalcification. The results of the DBCM are represented as a 3D plot of the permittivity against the time after recalcification and the frequency of the alternative current (**Fig 1A**). We compared two samples; normal blood and sufficiently heparinized blood. The heparinized blood sample did not form any blood clots at the end of the measurement. The DBCM analysis of the normal control blood exhibited a gradual increase in the dielectric permittivity at a frequency range between 2.5 MHz to 16 MHz. However, the heparinized sample exhibited no increase in the dielectric permittivity at that frequency range. Thus, we focused on the temporal change in the dielectric permittivity at 10 MHz (**Fig 1B**). The temporal change in the dielectric permittivity represented a sigmoidal increase in the normal blood sample, but that in the heparinized sample did not show any elevation of the permittivity. We calculated the derivative of the dielectric permittivity at this frequency, so that the derivative curve represented the temporal acceleration of the coagulation. The maximum value of the derivatives ranged between 0.012 and 0.044 (0.026 ± 0.007) in the normal samples with blood clot formation, however, that in the heparinized sample did not exceed 0.01 (0.004 ± 0.003), and it showed no overlap between the normal and heparinized blood samples (**Fig 1C**). These findings suggested that blood clot was generated when the maximum value of the derivative exceeded 0.01. We calculated the index representing coagulation, the end of acceleration time (EAT), defined as the time at which the derivative of the permittivity crossed 10% of the maximum value in its descending phase, only when the maximum value of the derivative exceeded 0.01 (**Fig 1B**).

We set the cutoff value for the EAT according to the following findings: several cases had a fluctuation in the derivative curve after the derivative returned back to zero, indicating completion of coagulation. We measured the maximum value of this fluctuation, and found it was 6.5% at the highest (**S1 Fig**). To avoid any contamination of this fluctuation, we defined the cutoff value of the EAT as 10% of the maximum.

### Reproducibility and interchangeability of the DBCM analysis

**Fig 2:**
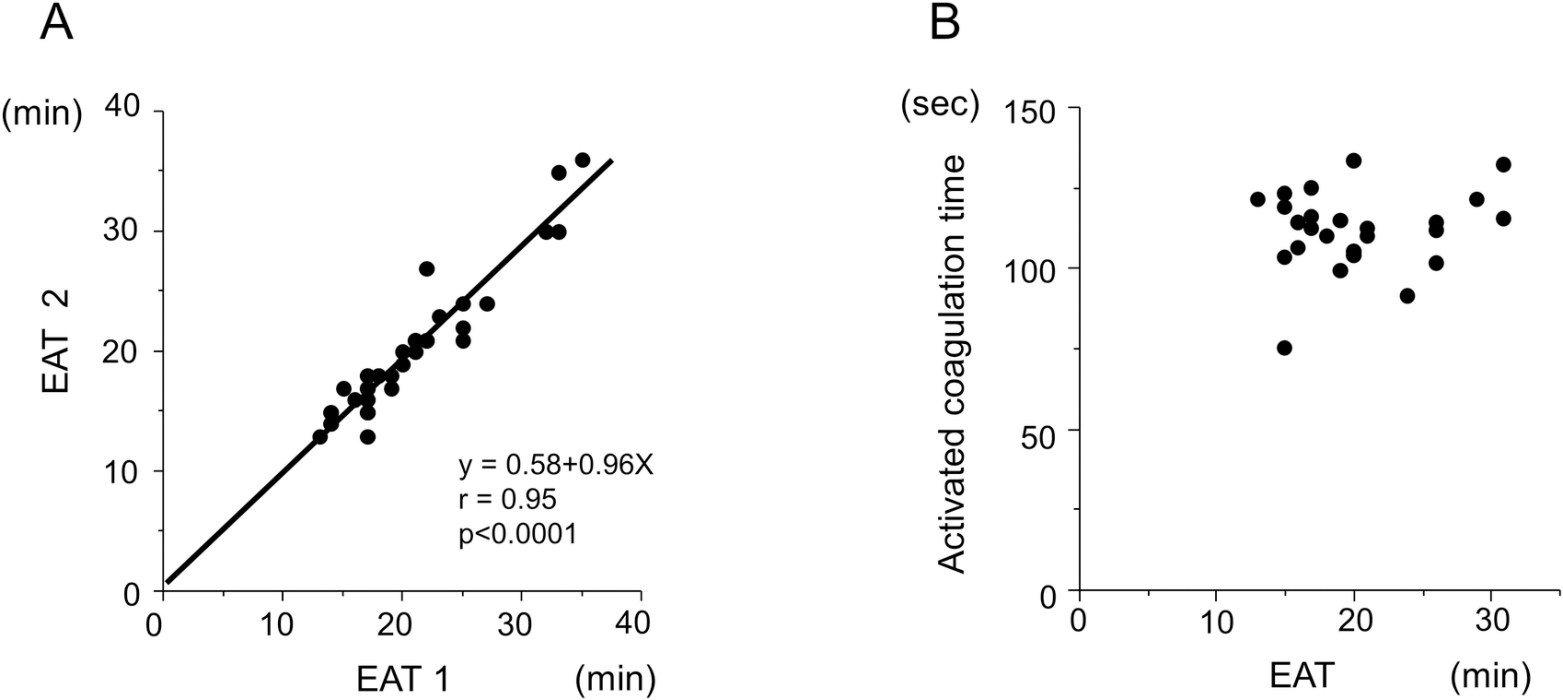
Reproducibility and interchangeability of the DBCM analysis. (A) The within-run reproducibility of the EAT is assessed by the correlation between the first measurement of the EAT (EAT1) and second one (EAT2) in healthy subjects (n = 40). The time interval between the first and second measurements was 3 to 5 minutes. The EAT at the time of the two measurements had a high reproducibility (y = 0.58 + 0.96x, r = 0.95, p <0.0001). (B) The interchangeability between the EAT and activated coagulation time (ACT) was evaluated by a simple regression analysis (n = 25). The EAT shows no significant correlation to the ACT.

Since whole blood coagulation assays are sensitive to the measurement condition, we evaluated the within-run reproducibility using the blood samples from healthy subjects. The EAT at the time of the two measurements had a high reproducibility, with an intraclass correlation coefficient of 0.964 (**Fig 2A**). We then examined the interchangeability between the EAT and activated coagulation time (ACT), the most popular conventional whole blood coagulation tests, using blood samples from healthy controls. In that comparison, the EAT did not show a significant correlation with the ACT (**Fig 2B**). We also tried to assess the correlation between the EAT and ACT using blood samples from patients who received intravenous heparin. However, the blood samples with an ACT >150 sec did not show any increase in the dielectric permittivity in the DBCM analysis as shown in Fig 1 within 60 min, and the EAT could not be conducted. It indicated the measurement range of the DBCM was narrower than that of the ACT.

### Measurement range of the coagulability using the DBCM analysis

**Fig 3:**
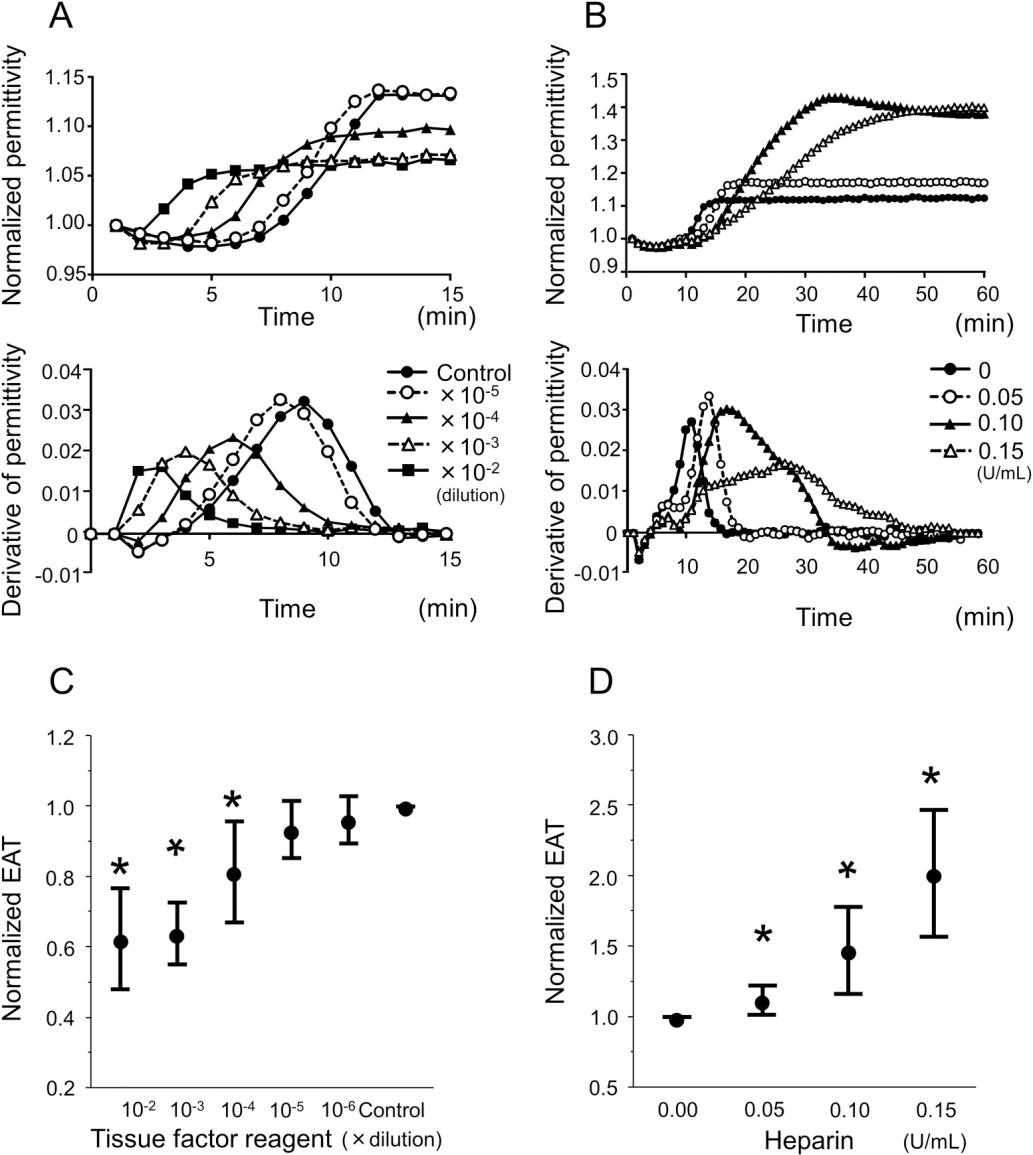
Dose-dependent change in the dielectric permittivity in response to TF reagent and heparin. (A and B) Representative traces of the temporal change in the normalized dielectric permittivity (upper panel) and its derivative (lower panel) at 10 MHz in a healthy subject. (A) The TF reagent was serially diluted and added to CaCl_2_. A lesser dilution of the TF reagent shifted the permittivity curve to the left in a dose-dependent manner, which means a larger amount of TF enhances the coagulation. The lower panel shows that the EAT, and the peak of the derivative also shifted to the left in a dose-dependent fashion. (B) A serial concentration of heparin is added to the citrated blood samples. A larger amount of heparin shifted the permittivity curve to the right in a dose-dependent fashion. (C) The EAT is normalized to the control conditions, and plotted against the serial dilution of the TF reagent. The normalized EAT shows a gradual shortening accompanied with a smaller dilution of the TF. (D) The EAT is normalized to the control sample, and plotted against the serial concentration of heparin, which shows dose-dependent prolongation. *, p <0.05 vs. control by paired *t* test.

To further delineate the measurement range of the coagulability that the DBCM could identify, we evaluated the EAT utilizing blood samples mixed with TF reagent or heparin. The addition of a serial dilution of TF reagent (10^−2^ to 10^−6^ dilution) into the blood samples shifted the permittivity curve to the left, which indicated a dose-dependent acceleration of the coagulation (**Fig 3A**). In contrast, the addition of a serial amount of heparin (0 to 0.15 U/ml) into the blood shifted the permittivity curve to the right, which represented a dose-dependent deceleration of the coagulation (**Fig 3B**). The relative change in the EAT was summarized by the dilution of the TF reagent, which showed a dose-dependent shortening, reaching a statistically significant difference when the amount of TF was between 10^−2^ to 10^−4^ (**Fig 3C**). On the other hand, the EAT revealed a dose-dependent increase with the addition of heparin of up to 0.15 U/ml (**Fig 3D**). We tried to assess the blood samples mixed with a higher concentration of heparin. However, the blood samples mixed with 0.20 U/ml of heparin showed neither an increase in the dielectric permittivity nor any visible blood clot formation after the recalcification within the observation period. A simultaneous measurement of the aPTT in these samples revealed it was >60 sec. These results indicated that the DBCM had the potential to assess the hypercoagulability for a mixture with a range of 10^−2^ to 10^−4^ of diluted TF, and to evaluate the hypocoagulability of up to 0.15 U/ml of heparin, which corresponded to aPTT of less than 60 sec.

We also evaluated the correlation between the EAT and aPTT using these samples mixed with heparin, which exhibited a strong positive correlation (**S2 Fig**). Thus the EAT had a certain interchangeability for aPTT within a limited measurement range.

### Change in the Coagulability in Patients with a high CHADS_2_ or CHA_2_DS_2_-Vasc score

Based on these fundamental assessments, we hypothesized that the DBCM was able to evaluate small changes in the coagulability in clinical samples. Thus, we performed a DBCM analysis in patients with different CHADS_2_ scores. The patients were divided into 3 groups according to their CHADS_2_ score (CHADS_2_ = 0, 1, and ≥2). The baseline characteristics of the study subjects are summarized in **Table 1**. The PT and aPTT were also measured in 49 cases, and the D-dimer in 20 cases.

**Table 1:** Characteristics of the patients classified by the CHADS_2_ score.

	CHADS_2_ = 0	CHADS_2_ = 1	CHADS_2_ ≥2	p value
n	42	22	20	
Female, n (%)	28 (67)	14 (64)	9 (45)	0.25
Age (mean ± SD)	38.0 ± 19.2	63.8 ± 12.8	74.9 ± 8.8	< .0001
CHF, n (%)	0 (0)	0 (0)	3 (15)	0.01
HT, n (%)	0 (0)	14 (64)	14 (70)	< .0001
Aged (≥75y), n (%)	0 (0)	3 (14)	14 (70)	< .0001
DM, n (%)	0 (0)	5 (23)	10 (50)	< .0001
Stroke/TIA, n (%)	0 (0)	0 (0)	4 (20)	0.002
β blockers, n (%)	1 (2)	3 (14)	4 (20)	0.07
Ca^2+^ blockers, n (%)	1 (2)	9 (41)	8 (40)	0.0001
ACE/ARB blockers, n (%)	0 (0)	4 (18)	12 (60)	< .0001
Antiplatelet drugs, n (%)	1 (2)	3 (14)	10 (50)	< .0001

CHF, congestive heart failure; HT, hypertension; DM, diabetes mellitus; TIA, transient ischemic attack

**Fig 4:**
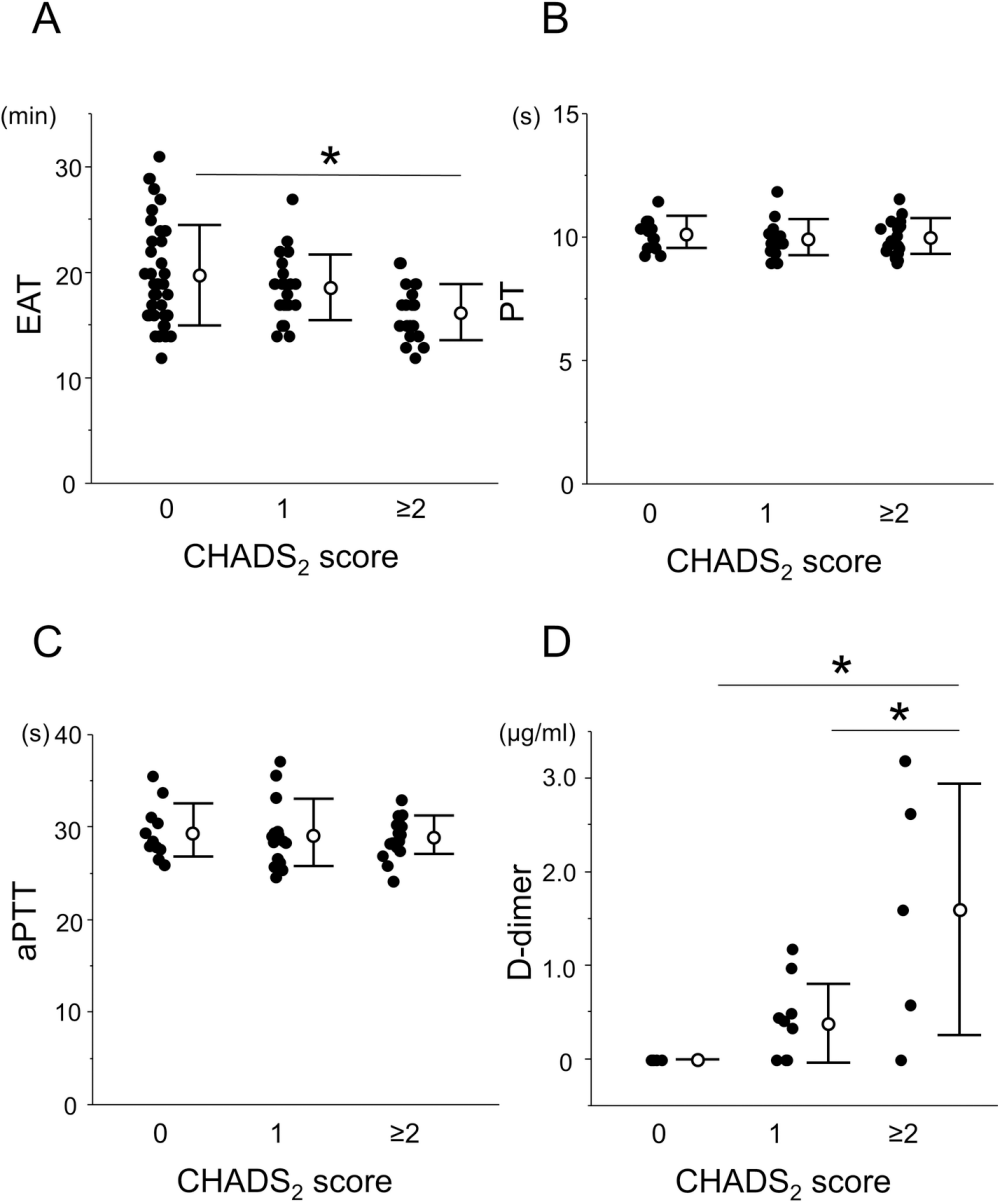
Coagulation parameters in the DBCM and conventional assays in groups with different CHADS_2_ scores. The patients were classified into 3 groups according to their CHADS_2_ score (0, 1, and ≥2) for a comparison with the EAT, (A), PT (B), aPTT (C) and D-dimer (D). The EAT and D-dimer showed a statistically significant difference in the 3 groups by ANOVA. Multiple comparisons revealed that the CHADS_2_ ≥2 group had a significantly shorter EAT than the CHADS_2_ = 0 groups and the CHADS_2_ ≥2 group had a significantly higher D-dimer level than the CHADS_2_ = 0 or 1 groups. The PT and aPTT exhibited no significant difference. *, p <0.05.

A comparison of the EAT among the 3 groups revealed that high CHADS_2_ scores were correlated with a shortening of the EAT (19.8 ± 4.8, 18.6 ± 3.1, and 16.3 ± 2.7 min in CHADS_2_ = 0, 1, and ≥2 groups, respectively, p = 0.0065 by ANOVA). Multiple comparisons revealed that the CHADS_2_ ≥2 group had a significantly shorter EAT than the CHADS_2_ = 0 group (**Fig 4A**). We also compared the PT and aPTT in these groups. There was no significant difference in the PT (10.3 ± 0.7, 10.0 ± 0.7, and 10.1 ± 0.7 sec for the CHADS_2_ = 0, 1, and ≥2 groups, respectively, p = 0.734 by ANOVA), and aPTT (29.8 ± 2.9, 29.5 ± 3.6, and 29.3 ± 2.1 sec for the CHADS_2_ = 0, 1, and ≥2 groups, respectively, p = 0.893 by ANOVA) among the 3 groups (**Fig 4B and 4C**). However, the CHADS_2_ ≥2 group had a significantly higher D-dimer level than the CHADS_2_ = 0 or 1 groups (0.00 ± 0.00, 0.39 ± 0.42, and 1.60 ± 1.34 μg/ml in CHADS_2_ = 0, 1, and ≥2 groups, respectively, p = 0.006 by ANOVA) (**Fig 4D**).

**Fig 5:**
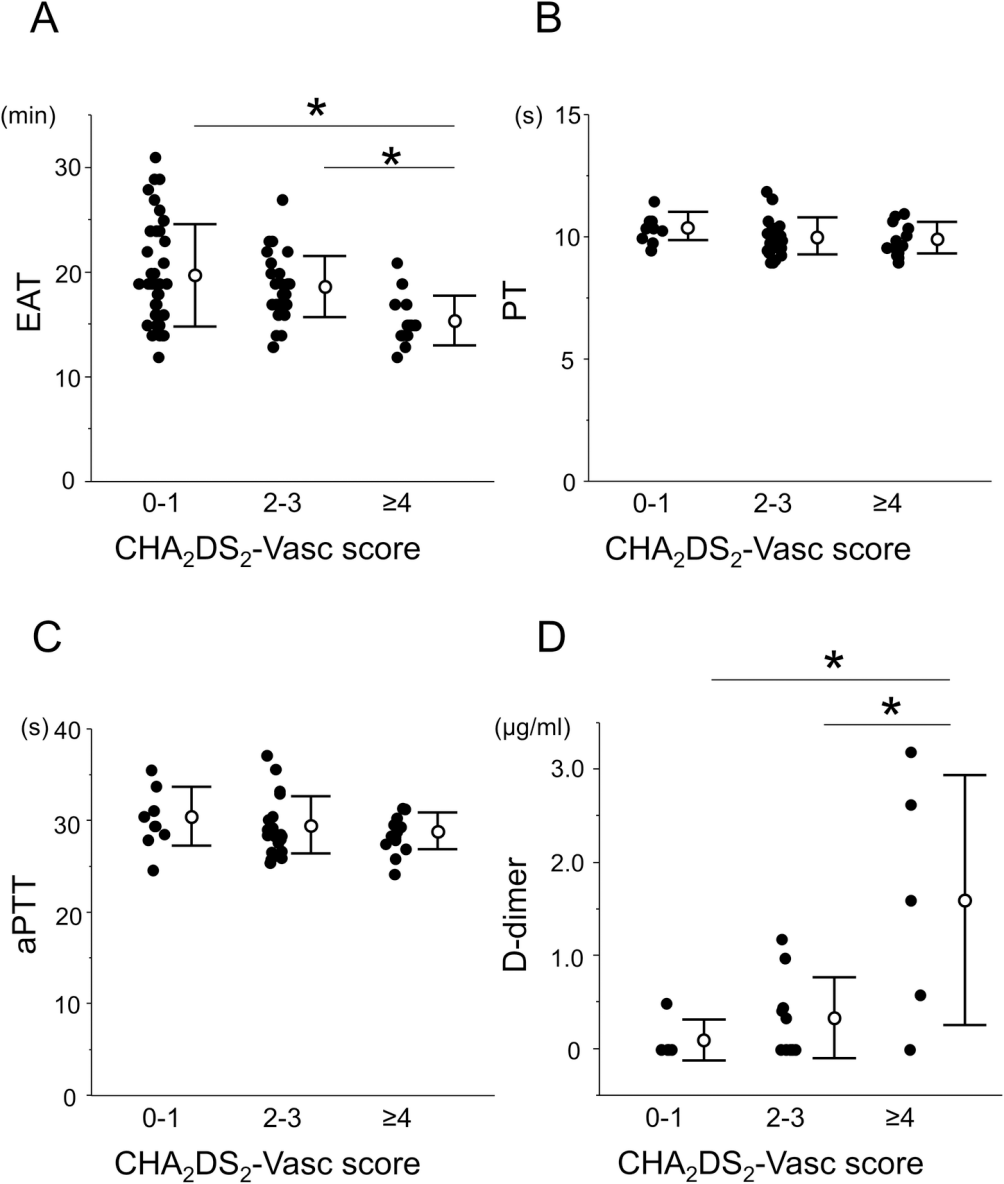
Coagulation parameters in the DBCM and conventional assays in groups with different CHA_2_DS_2_-Vasc scores. The patients were classified into 3 groups according to their CHA_2_DS_2_-Vasc score (0–1, 2–3, and ≥4) for a comparison with the EAT (A), PT (B), aPTT (C) and D-dimer (D). The EAT and D-dimer showed a significant difference by ANOVA, and multiple comparisons revealed that the CHA_2_DS_2_-Vasc score ≥4 group had a significantly shorter EAT and higher D-dimer than the other 2 groups. Neither the PT nor aPTT exhibited any difference among the 3 groups. *, p <0.05.

The CHA_2_DS_2_-Vasc score was also utilized to stratify the risk of a stroke. Thus, the study group was evaluated for the CHA_2_DS_2_-Vasc score. Due to a shortage of the number of subjects, we divided the subjects into 3 groups (CHA_2_DS_2_-Vasc = 0–1, 2–3, and ≥4) (**Table 2**). Similarly for the comparison in the CHADS_2_ score group, the higher CHA_2_DS_2_-Vasc score group had an increased coagulability represented by a shortening of the EAT (19.7 ± 4.9, 18.6 ± 2.9, and 15.4 ± 2.4 min for CHA_2_DS_2_-Vasc score = 0–1, 2–3, and ≥4 groups, respectively, p = 0.0029 by ANOVA). Multiple comparisons revealed that the CHA_2_DS_2_-Vasc ≥4 group had a significantly shorter EAT than both the CHA_2_DS_2_-Vasc = 0–1, and 2–3 groups. However, the PT and aPTT exhibited no differences among the 3 groups (PT: 10.5 ± 0.6, 10.1 ± 0.8, and 10.0 ± 0.6 sec in CHA_2_DS_2_-Vasc = 0–1, 2–3, and ≥4 groups, respectively, p = 0.238 by ANOVA, and aPTT: 30.5 ± 3.2, 29.5 ± 3.1, and 28.9 ± 2.0 sec for the CHA_2_DS_2_-Vasc = 0–1, 2–3, and ≥4 groups, respectively, p = 0.428 by ANOVA). In a similar fashion as the CHADS_2_ score, D-dimer level in the CHA_2_DS_2_-Vasc ≥4 group was significantly higher than that in the CHA_2_DS_2_-Vasc = 0–1 or 2–3 groups (0.10 ± 0.22, 0.34 ± 0.44, and 1.60 ± 1.34 μg/ml for the CHA_2_DS_2_-Vasc score = 0–1, 2–3, and ≥4 groups, respectively, p = 0.008 by ANOVA) (**Fig 5**).

**Table 2:** Characteristics of the patients classified by the CHA_2_DS_2_-Vasc score.

	CHA_2_DS_2_-Vasc = 0–1	CHA_2_DS_2_-Vasc = 2–3	CHA_2_DS_2_-Vasc ≥4	p value
n	39	31	14	
Female, n (%)	22 (56)	19 (61)	10 (71)	0.60
Age (mean ± SD)	34.7 ± 16.2	66.5 ± 12.1	77.2 ± 5.4	< .0001
CHF, n (%)	0 (0)	0 (0)	3 (21)	0.004
HT, n (%)	2 (5)	16 (52)	10 (71)	< .0001
Aged (≥75y), n (%)	0 (0)	7 (23)	10 (71)	< .0001
Aged (65~74y), n (%)	3 (8)	15 (48)	4 (29)	0.0004
DM, n (%)	1 (3)	7 (23)	7 (50)	0.0002
Stroke/TIA, n (%)	0 (0)	1 (3)	3 (21)	0.01
Vascular disease, n (%)	0 (0)	0 (0)	4 (29)	0.0005
β blockers, n (%)	2 (5)	3 (10)	3 (21)	0.20
Ca^2+^ blockers, n (%)	3 (8)	10 (32)	5 (36)	0.016
ACE/ARB blockers, n (%)	0 (0)	8 (26)	8 (57)	< .0001
Antiplatelet drugs, n (%)	0 (0)	5 (16)	9 (64)	< .0001

CHF, congestive heart failure; HT, hypertension; DM, diabetes mellitus; TIA, transient ischemic attack

### Correlation between the DBCM analysis and conventional coagulation assays

**Fig 6:**
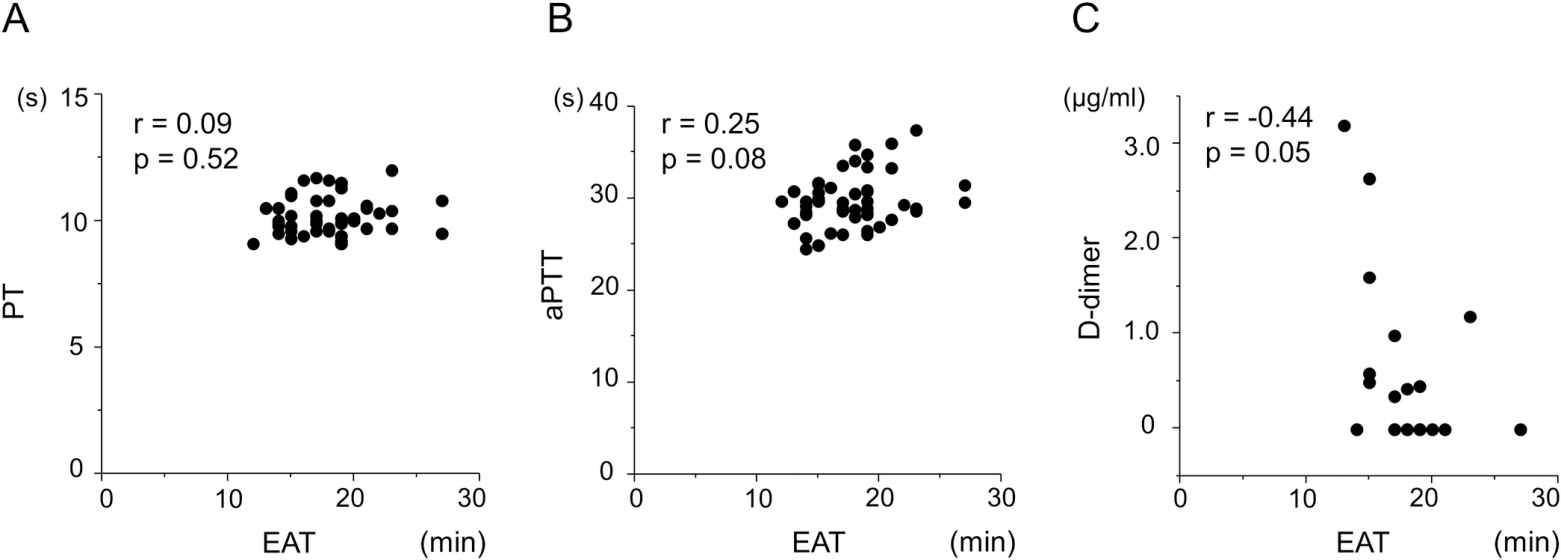
Correlation between the EAT and PT, aPTT, and D-dimer. Scatter-plots are shown between the EAT and PT (A), aPTT (B), and D-dimer (C). All of them exhibited no statistically significant correlations.

Then we evaluated the correlation between the EAT and conventional coagulation assays in a subgroup of patients. The EAT had no significant correlation to PT, and tended to have a weak correlation to aPTT, without any statistical significance. We also found that D-dimer level tended to be high in shorter EAT patients, but it also did not reach statistical significance (**Fig 6**).

### The effect of antiplatelet and anticoagulation on the DBCM analysis

In this study, we did not exclude any cases receiving antiplatelet drugs (Tables 1 and 2). Since the CHADS_2_ = 0 group had only 1 patient taking an antiplatelet drug, we performed subanalyses to evaluate the effect of antiplatelets in the CHADS_2_ = 1 and ≥2 groups. The EAT exhibited no differences between the groups with or without antiplatelet drugs (17.7 ± 4.0 vs. 18.7 ± 3.1 min, 16.4 ± 2.3 vs. 16.1 ± 3.1 min, in CHADS_2_ = 1 and ≥2 groups, respectively).

**Fig 7:**
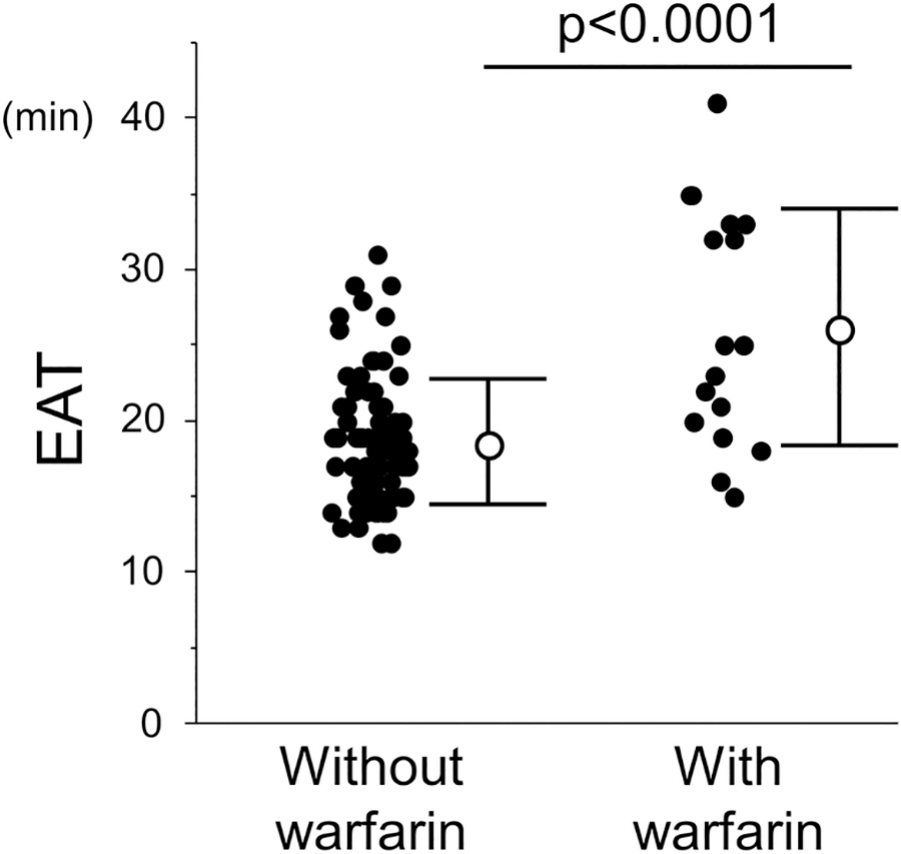
Comparison of the EAT between patients with and without receiving warfarin. The EAT was significantly prolonged in the group receiving warfarin.

The EAT was also measured in patients who received warfarin, to investigate if the EAT could assess the effect of anticoagulation. The EAT in the warfarin group was significantly longer than that in those without warfarin. The assessment of the correlation between PT and the EAT in patients with or without warfarin showed a significant positive correlation (**Fig 7 and S3 Fig**).

## Discussion

### Main findings

This study demonstrated that the DBCM had the potential to detect small changes in the whole blood coagulability. Although the theoretical utility of the DBCM was shown in previous papers [18, 19], to the best of our knowledge, this report is the first to show the usefulness of the DBCM for evaluating the whole blood coagulability by a systematic analysis. With a comparison between normal blood and sufficiently heparinized blood that failed to form blood clots, we found that the temporal change in the dielectric permittivity at 10 MHz was suitable for delineating the change in the blood clot formation. We identified a novel parameter for coagulation, the EAT from the derivative of the temporal change in the dielectric permittivity. The EAT showed a dose-dependent shortening with the addition of diluted TF reagent, and also exhibited a dose-dependent prolongation with the addition of heparin. In the clinical setting, the EAT revealed an increased coagulability in high CHADS_2_ or CHA_2_DS_2_-Vasc score patients without AF.

### Coagulation parameters derived from the DBCM

The clinical utility of the DBCM including the establishment of an adequate index for coagulation has not been achieved. In this study, we defined the EAT as a novel index for coagulation. The EAT could detect small changes both in the hyper- and hypo-coagulation, and the measurement range of the EAT was between dilutions of the TF reagent of ×10^−2^ to ×10^−4^ for the hypercoagulability, and between concentrations of heparin of 0 and 0.15 U/ml for the hypocoagulability.

The EAT had a strong correlation with aPTT in heparinized blood samples, indicating that the EAT had a potential to assess reduced coagulability similar to aPTT. However, the DBCM failed to exhibit an increase in the dielectric permittivity in the heparinized blood with an aPTT of >60 sec, because the DBCM analysis did not use any procoagulant reagents.

The striking finding in this study was that the EAT could detect the different coagulabilities in blood with a highly diluted TF reagent. Thus the measurement range of the DBCM was narrower and shifted to hypercoagulability, compared with that of the conventional coagulation tests. However, within this measurement range, the DBCM had the potential to quantify the coagulability with a certain sensitivity. This difference in the sensitivity and measurement range may explain why the EAT had a significant correlation with aPTT and PT in subjects with anticoagulation, but not in those subjects without anticoagulation.

### Whole blood coagulation assay

Recent findings revealed that the cell surface played a pivotal role in the coagulation process [20]. In contrast to most conventional coagulation assays utilized for plasma, the whole blood assay has the advantage of being able to assess the coagulability including the red blood cell and platelet function. To date, several whole blood coagulation assays have been established. The ACT is widely used for point-of-care testing during cardiopulmonary bypass operations and cardiac catheterization procedures, including percutaneous coronary intervention and catheter ablation [21]. However, it is also well known that the ACT has a large variability and a relatively low reproducibility [22]. In addition, the ACT assay utilizes native blood samples, thus an immediate measurement after the collection of the blood is required. In contrast, the DBCM does not require an immediate measurement, because it assesses the permittivity change after recalcification using citrated blood samples. The EAT also has an advantage of a relatively high within-run reproducibility. However, the EAT could be derived from a blood sample with an ACT of less than 150 seconds. These findings indicate that the DBCM may be a more convenient and reliable coagulation index than the ACT with a limited measurement range.

In this study, the EAT failed to show any significant correlation to ACT. This result was explained by same reason for the loss of the correlation between the EAT and aPTT. Although ACT has an advantage of evaluating anticoagulated blood with heparin, we compared the EAT and ACT using blood samples from subjects without anticoagulants. In this group of subjects, the sensitivity may be lower in ACT than the EAT. A large variability and lesser reproducibility of ACT also contributed to the loss of a correlation.

Thromboelastography (TEG) and rotational thromboelastometry (ROTEM) have become popular monitoring modalities to assess whole blood coagulability, by measuring the viscoelastic changes under low-shear stress conditions. Recent reports described that ROTEM had the potential to detect the hypercoagulability in patients with lung cancer or obesity [23, 24]. Further analysis will be required to elucidate the interchangeability between the DBCM and these viscoelastic evaluations.

### CHADS_2_ score and hypercoagulability

As aforementioned, the CHADS_2_ score or CHA_2_DS_2_-Vasc score are useful for the risk stratification of strokes in cases with AF [2, 3]. This study demonstrated the correlation between a higher CHADS_2_ score or CHA_2_DS_2_-Vasc score and the whole blood hypercoagulability detected by the DBCM. Although previous studies have indicated that each component of the CHADS_2_ score is related to an increased coagulation separately [11–17], this study suggested that each component may have an additive effect on the hypercoagulability. We enrolled patients without AF to avoid the effect of anticoagulation in this study. Since previous studies showed that AF itself enhanced the coagulability of blood [25, 26], the DBCM assessment may demonstrate a more distinct difference in AF patients.

However, the EAT exhibited no significant difference between the CHADS_2_ = 1 group and CHADS_2_ = 0 group. It indicated that only 1 risk factor may be insufficient to increase the coagulability significantly. This finding is in line with the clinical observation that a CHADS_2_ = 1 is a modest risk for a stroke in patients even with AF. The incidence of a stroke with a CHADS_2_ = 0 was 0.8% to 1.9% and that with a CHADS_2_ = 1 was 2.2% to 2.9% per year in patients with AF [3, 27, 28], and 0.3% and 1.1% in patients without AF [5]. Further, it is difficult to find high risk patients with the present CHADS_2_ scoring strategy. Intriguingly, we found that the EAT had a large variation in the CHADS_2_ = 0 and 1 groups. The subjects with a shorter EAT might have hypercoagulability, and have a higher risk of thrombosis in spite of their low CHADS_2_ scores. It suggested that the DBCM analysis may provide additional information for the risk stratification in low CHADS_2_ score groups. However, we need to perform a prospective study to prove our hypothesis.

It has been described that shortening of aPTT is also considered as a marker of hypercoagulability in patients with deep vein thrombosis [29, 30], and diabetes [15]. As other biomarkers representing thrombus formation and fibrinolysis, several studies have revealed an elevated D-dimer level, thrombin-antithrombin complex, or prothrombin fragment 1+2 in patients with heart failure [15, 31] and diabetes [32]. Our results were partly consistent with the results of the previous reports. The D-dimer level in the CHADS_2_ ≥2 group was significantly higher than that in the CHADS_2_ = 0 or 1 groups. In this study, however, aPTT exhibited no significant change among the different CHADS_2_ or CHA_2_DS_2_-Vasc score groups. The discrepancy may be caused by the inclusion criteria and/or number of subjects. A large scale study will answer this point in the future.

### Effect of antiplatelet drugs

In contrast to the anticoagulants, we did not exclude any of the patients taking antiplatelets in this study. When we compared the EAT between the patients with or without antiplatelet drugs in the respective CHADS_2_ groups, there were no significant differences. It indicated that the influence of antiplatelet drugs was relatively small in the EAT. However, a systematic large scale evaluation will be required to assess the contribution of antiplatelets in the DBCM analysis in the future.

### Study limitations

Since this was the proof of the concept of this study, the number of subjects was relatively small. Secondly, we showed that the EAT became shortened in the higher CHADS_2_ or CHA_2_DS_2_-Vasc score groups in this study. However, it is still undetermined whether or not this assessment can predict the risk of thromboembolisms in the clinical setting. A large scale prospective study will be required to prove the clinical utility of the DBCM.

## Conclusions

The DBCM, a novel highly sensitive measurement method for whole blood coagulation, can identify small changes in the coagulation status. Patients with higher CHADS_2_ or CHA_2_DS_2_-Vasc scores exhibited hypercoagulability without AF.

## Supporting Information

S1 Fig The definition of the EAT. (A)Representative trace of normalized permittivity (%) at 10 MHz. There was fluctuation after completion of coagulation. (B)We measured the maximum value of fluctuation (2.3 ± 2.2%), which was 6.5% at highest.
(TIF)

S2 Fig Correlation EAT and aPTT in blood samples mixed with serial concentration of heparin. The EAT and aPTT showed strong positive correlation.
(TIF)

S3 Fig Correlation between the EAT and PT in patients with and without warfarin. The EAT and PT showed significant positive correlation.
(TIF)
