# Supplementary figures and images for: Novel Dielectric Coagulometer Identifies Hypercoagulability in Patients with a High CHADS2 Score without Atrial Fibrillation
Source: PLoS One. 2016 Jun 8;11(6):e0156557. doi: 10.1371/journal.pone.0156557 (PMC4898832; doi:10.1371/journal.pone.0156557)

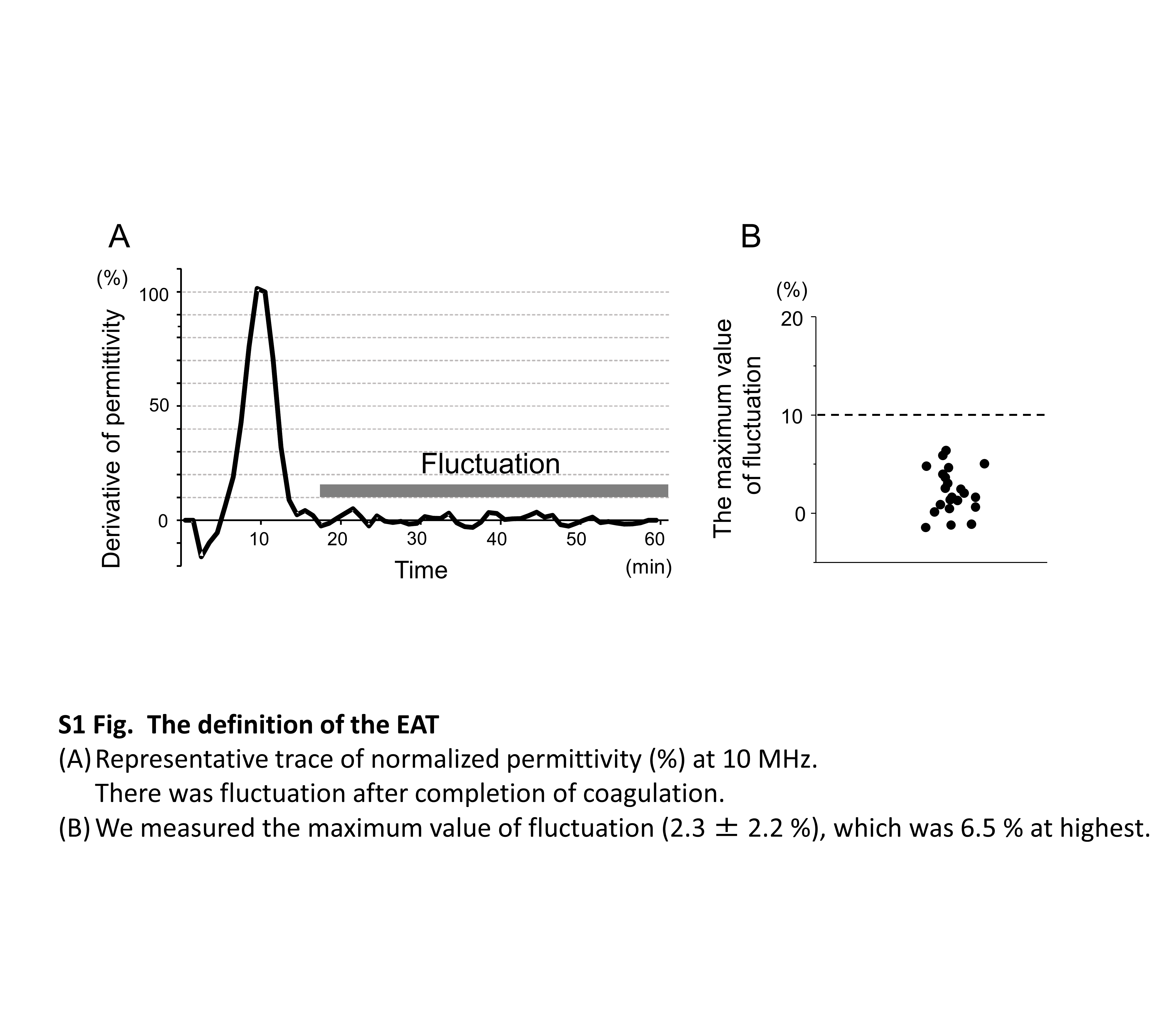

Supplement: S1 Fig — (A)Representative trace of normalized permittivity (%) at 10 MHz. There was fluctuation after completion of coagulation. (B)We measured the maximum value of fluctuation (2.3 ± 2.2%), which was 6.5% at highest. (TIF) [file pone.0156557.s001.tif]

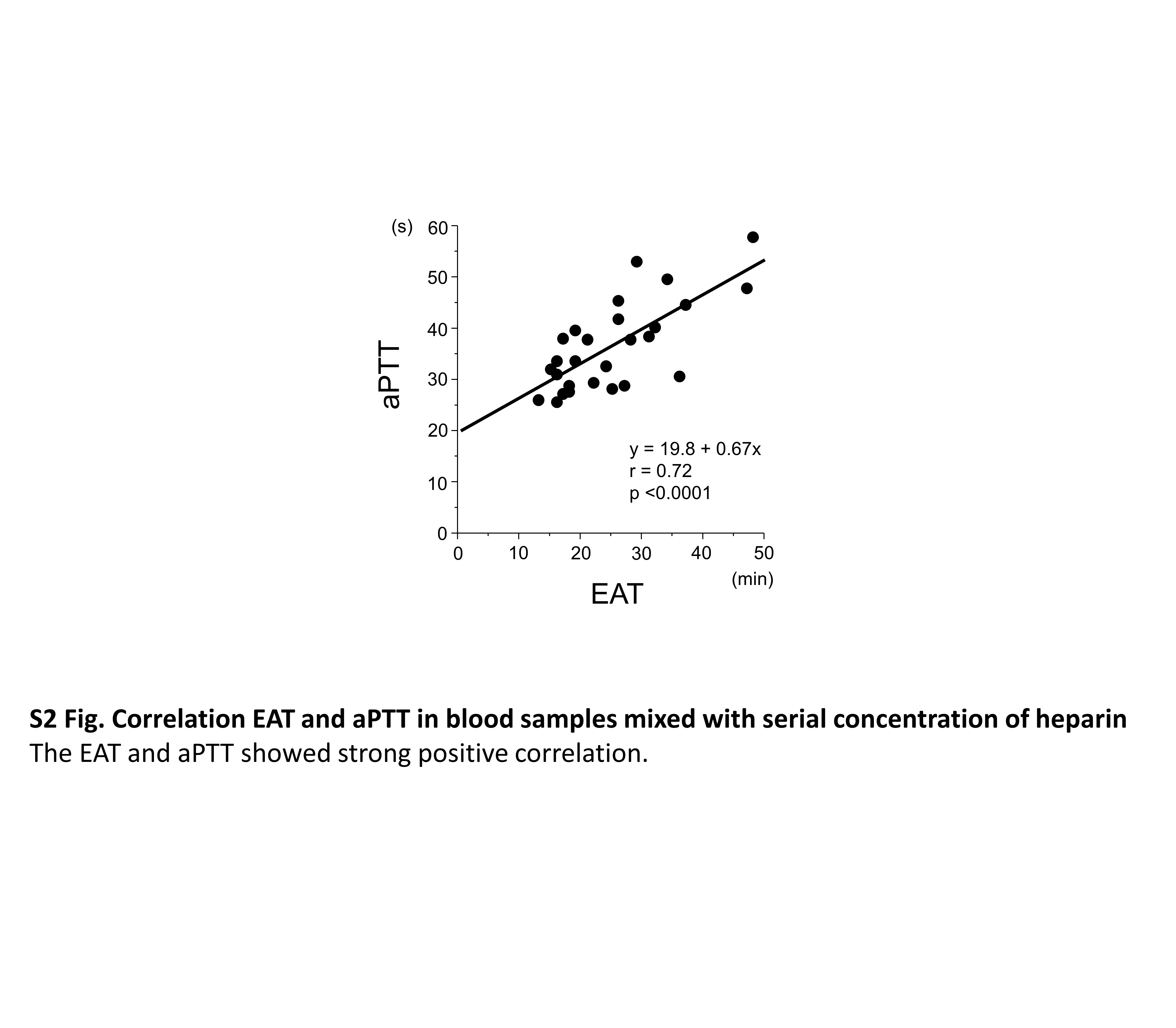

Supplement: S2 Fig — The EAT and aPTT showed strong positive correlation. (TIF) [file pone.0156557.s002.tif]

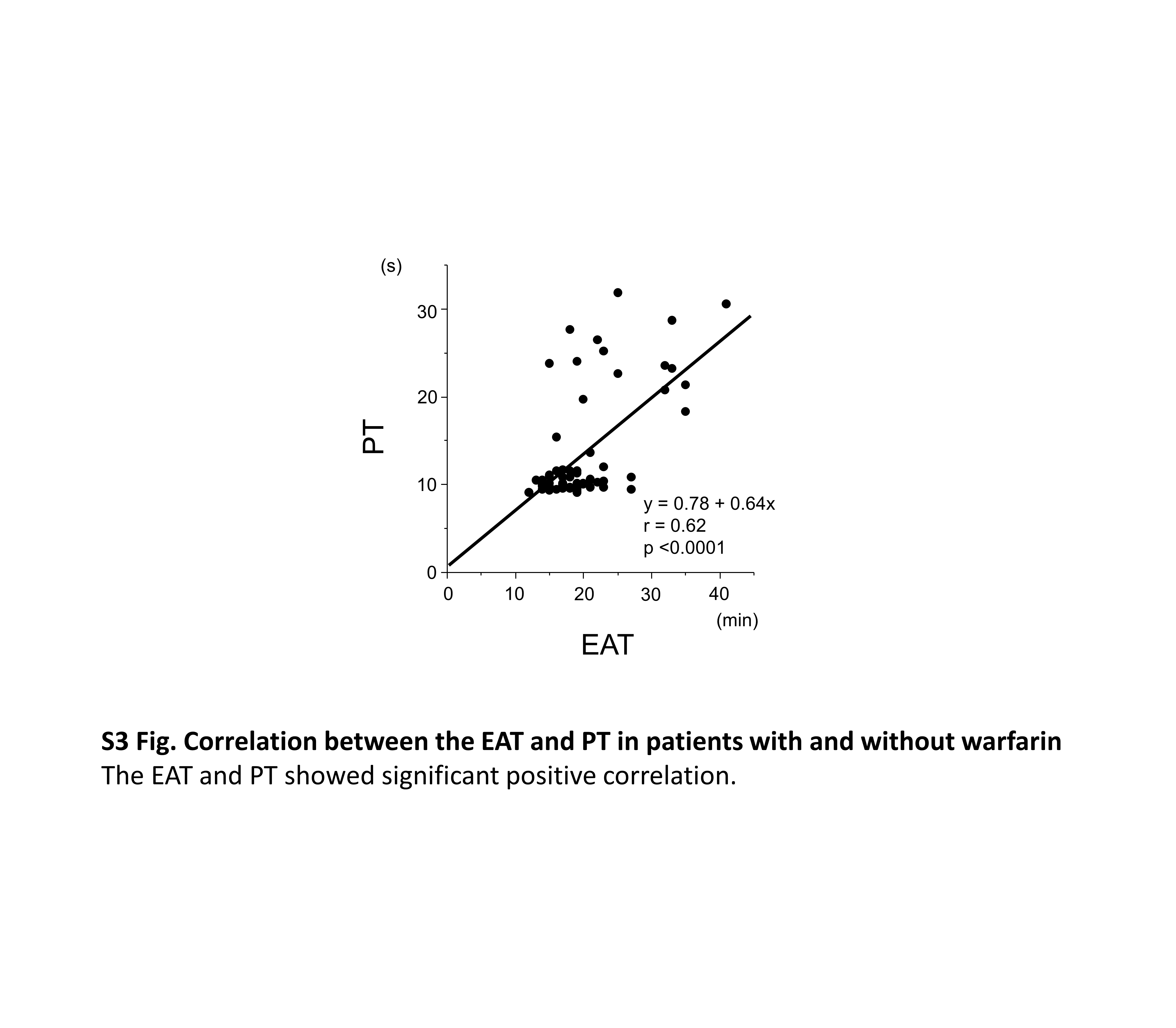

Supplement: S3 Fig — The EAT and PT showed significant positive correlation. (TIF) [file pone.0156557.s003.tif]
